# The Effect of Physical Activity on Neurotrophin Concentrations and Cognitive Control in Patients With a Depressive Episode

**DOI:** 10.3389/fpsyt.2022.777394

**Published:** 2022-04-25

**Authors:** Anna Jasińska-Mikołajczyk, Katarzyna Drews, Katarzyna Domaszewska, Grzegorz Kolasa, Marta Konofalska, Katarzyna Jowik, Maria Skibińska, Filip Rybakowski

**Affiliations:** ^1^Department of Adult Psychiatry, Poznan University of Medical Sciences, Poznan, Poland; ^2^Department of Physiology and Biochemistry, Poznan University of Physical Education, Poznan, Poland; ^3^Department of Psychiatric Genetics, Poznan University of Medical Sciences, Poznan, Poland

**Keywords:** neuroplasticity, BDNF, Stroop test, depression, exercise

## Abstract

**Background:**

Cognitive deficits occur in most patients with affective disorders. The role of neurotrophic factors (e.g., BDNF) as modulators of brain plasticity affecting neurocognitive abilities has been emphasized. Neurotrophin concentrations may change under the influence of various interventions, including physical activity. Selected studies have shown that cognitive function may also be affected by exercise.

**Aim:**

The aim of the study was to determine whether physical activity changes the concentration of neurotrophins and their receptors in patients with an episode of depression. It was also examined how one session of aerobic exercise affects cognitive control.

**Methods:**

The study included 41 participants. The subjects were asked to exercise on a cycloergometer for 40 min with individually selected exercise loads (70% VO_2_max). Before and shortly after the exercise blood samples were acquired to perform blood assays (proBDNF, BDNF, TrkB, NGFR). The participants also performed a Stroop test twice—before the exercise and 10 min after its cessation.

**Results:**

The single bout of physical exercise did not cause any significant changes in the concentration of neurotrophic factors. The SCWT results: both the mean reading time (29.3 s vs. 47.8 s) and the color naming time (36.7 s vs. 50.7 s) increased. The patients made more mistakes after physical exercise, both in part A (0.2 vs. 1.5) and B (0.6 vs. 1.5). The so-called interference effect decreased—the difference between naming and reading times was smaller after exercise (6.2 s vs. 2.4 s). No significant correlations were found between the concentrations of the studied neurotrophic factors and the Stroop test results.

**Conclusions:**

The results did not confirm changes in neurotrophin concentration under the influence of a single session of physical activity. The shortening of the interference time after exercise may be caused by practice effects. A significant limitation of the study is the use of the Stroop test twice in short intervals.

## Introduction

### Affective Disorders and Cognitive Impairments

Depression affects more than 300 million people worldwide (1). Bipolar disorder affects about 45 million people worldwide (1) and it consists of both depressive and maniac episodes separated by periods of normal mood. Affective disorders involve cognitive impairments. Depressed patients frequently report difficulties with concentrating their attention and experience problems with tasks requiring prolonged focus (2). The pace of work and the ability to learn deteriorate as well. Depression also negatively affects psychomotor speed (i.e., coordination, dexterity, acting with appropriate speed and force), which involves both cognitive and physical abilities (3, 4). For proper cognitive functioning, it is important that the course of executive functions (forming concepts, starting and continuing actions, understanding sets and selecting information depending on the situation, storing information about the criteria of current actions and switching to new rules of action, solving problems, and making decisions) remains undisturbed (5–7). In 20–30% of patients with depression, deficits have been described in this area as well (2, 5). Some papers draw attention to the fact that cognitive impairments (deficits in selective attention, working memory, and long-term memory) continue to persist during remission and that improvement in mood is not always accompanied by normalization of cognitive abilities (8). Data from meta-analyses confirm that patients with bipolar disorder also have neurocognitive dysfunction (9–11).

### Neurogenesis and Neuroplasticity

The human brain retains plasticity as its nerve cells retain the ability to change both their structure and function throughout the life of an individual. Interactions with the environment cause existing neuronal networks to change, and new synaptic connections are formed. Modulation of neurogenesis is associated with numerous factors acting at different stages of neuronal formation and differentiation. The best known neurotrophins include nerve growth factor (NGF), brain-derived neurotrophic factor (BDNF), neurotrophin 3 (NT-3), and neurotrophin 4 (NT-4).

Brain-derived neurotrophic factor (BDNF) has been found in almost all brain regions of the adult central nervous system. Expression of BDNF and its receptor, TrkB (tropomyosin receptor kinase B), has been observed in skeletal muscles, cardiac muscles, liver, and fat cells (12). BDNF is released from the brain into the blood both at rest (13) and during exercise (14). Like other neurotrophins, BDNF is initially synthesized as a precursor protein (proBDNF). ProBDNF and mBDNF exert opposite biological effects by activating two distinct receptor systems (15). Activation of the TrkB receptor prevents apoptosis and neurodegeneration, whereas activation of the p75NTR receptor initiates cell death (16). It plays an important role in the modulation of synaptic transmission and plasticity, which determines the adaptation of the nervous system to constant environmental changes. It supports cognitive processes such as learning and memory by modulating long-term potentiation (LTP) and depression (LTD) in hippocampal synapses (17, 18). The p75NTR receptor, also known as the nerve growth factor receptor (NGFR), was the first discovered member of the tumor necrosis factor receptor (TNFR) superfamily, with low affinity for NGF. NGFR affects cell cycle regulation through its function as a suppressor of certain processes in progenitor and somatic cells (19, 20).

### Neuroplasticity and the Role of BDNF in Affective Disorders

Depression may correspond to a significant slowing down or inhibition of neuroplasticity. Changes in the expression of genes regulating brain plasticity and reduced numbers of interneuronal connections have been observed; a decrease in *de novo* neurogenesis has also been postulated (21). It has been shown that the concentration of this neurotrophin in depressed patients differs from the healthy population both before treatment (effect size 0.91, 95% CI 0.70–1.11) and after treatment (effect size 0.34, 95% CI 0.02–0.66) (22). BDNF levels increase after treatment (effect size 0.62, 95% CI 0.36–0.88), and this increase correlates with improvement in depression scores changes (*p* = 0.02) (22). Plasma BDNF concentration is also lower in patients with bipolar disorder (Hedges' g = −0.28, 95% CI: −0.51 to −0.04, *p* = 0.02) (23).

### Physical Activity in Affective Disorders

Physical activity has a positive effect on both the somatic and mental state. It reduces the risk of developing depressive disorders in all age groups. A meta-analysis by Schuch found that exercise has a large and significant antidepressant effect in people with depression (including MDD) (24). There are also contradictory results. No improvement in the quality of life, reduction in depressive symptoms or the relationship between exercise and achieving remission (25) was noted. It is not entirely clear how exercise could change the course of mental illness. One of the proposed mechanisms is change in neuroplasticity induced by physical activity. It was confirmed that both a single exercise and regular physical activity increase the concentration of BDNF (26, 27). The increase in BDNF levels after exercise was observed in a study conducted in young, healthy participants (28, 29). A significant increase in BDNF was found also in patients with depression (30, 31). It was noted that changes in the concentration of neurotrophins may be associated with changes in selected cognitive functions (32). However, some studies show that the level of BDNF under the influence of physical activity, especially in patients with depression, does not change significantly. In one meta-analysis (199 patients, only aerobic exercise of various duration), no significant change in BDNF concentration was observed after exercise (*p* = 0.75) (33).

The effect of exercise on cognition, especially among patients with depressive disorders, is inconclusive. A review of the available literature conducted by Brondino et al. (34) found no evidence for beneficial effects of physical activity on cognitive function. The authors found no change in either overall cognitive performance or its individual components (speed of processing, attention/vigilance, working memory, verbal and visual memory, and reasoning), regardless of the duration and intensity of exercise. No significant effect of PA on cognitive performance was found, but the results pertaining to several neurocognitive functions (not including verbal memory and verbal fluency/working memory) appeared to be better among patients engaging in PA than in the sertraline group; however, they did not differ from the results achieved in the placebo group (35). One of the studies found that the aerobic training improved depressive symptoms and cognitive control processes in individuals with MDD (36).

Fewer studies are available on the impact of a single bout of physical activity on cognitive performance in depressed patients. One analysis (79 studies, 2,072 subjects) conducted by Chang showed that a single bout of exercise (aerobic, non-aerobic, and muscular resistance) had a positive effect on cognitive function, but the effects observed during the exercise, immediately afterwards, and at a later time were small (37). Improvements after a single session of exercise were observed, among others, in the TREAD (Treatment with Exercise Augmentation for Depression) study. High-intensity exercise was associated with improved spatial working memory, while other cognitive functions (including psychomotor speed, attention, visual memory, and spatial planning) improved regardless of task intensity (38). The authors postulated that physical activity may be a beneficial intervention in depressed patients complaining of cognitive decline, especially if the exercise regime conforms to WHO guidelines. Improvements in some cognitive functions, primarily related to the prefrontal cortex (i.e., working memory, verbal fluency, speed of information processing), were observed after a single session of high-intensity exercise (39).

Many studies linking physical activity and cognitive function have been conducted in healthy populations. Due to the physiological decline in cognitive performance that progresses with age, these experiments have often featured older adults. Physical activity has been found to primarily affect working memory and executive functions, with effects lasting up to ~2 h after cessation of exercise (40). The most commonly used tools (tests) have been those that assess skills depending on the functioning of the prefrontal cortex (attention and perception tasks that focus on reaction time and on verbal and visual working memory). In a review conducted by Basso and Suzuki (40), the authors found that a single session of exercise primarily affects executive functions, including attention, working memory, problem solving, cognitive flexibility, verbal fluency, decision making, and inhibitory control. Selected publications have also made the suggestion that the intensity of the physical activity used may be a differentiating factor (41). The researchers concluded that an increase in BDNF after exercise is likely to be of peripheral (rather than central) origin, and they noted an association between the post-intervention BDNF levels and cognitive function. Acute changes in peripheral BDNF following physical exercise were demonstrated, and individuals with greater increases in plasma BDNF following physical exercise exhibited greater cognitive training gains, but only if the cognitive training session was preceded as opposed to followed by physical exercise (42).

### Aim

In the present study, we attempted to evaluate the effects of exercise on neurotrophic factor levels in depressed individuals. We examined whether a single session of aerobic exercise would change the levels of BDNF and other proteins (proBDNF, TrkB, p53). The influence of exercise on selected cognitive abilities (primarily cognitive control) of patients was also assessed using a Stroop test.

## Materials and Methods

### Participants

Adult (18–65 years, both sexes: 24 women and 17 men) inpatients diagnosed with major depressive disorder (MDD) or a depressive episode in the course of bipolar disorder (BD) using semi-structured interviews based on ICD-10 (International Classification of Diseases) and hospitalized in a psychiatric clinic in Poznań were invited to participate in the study. The diagnoses were made by two psychiatrists. The severity of depression was assessed using the Hamilton Depression Rating Scale (HDRS). The exclusion criteria included the occurrence of an acute psychotic episode, a comorbid diagnosis of substance abuse during the previous 6 months, as well as severe cardiovascular and skeletomuscular disorders (constituting a contraindication to participate in physical exercise). All the patients were examined by a cardiologist before the study. They all provided their written consent after being informed about the study methods. The study was approved by the Ethical Committee of the Poznan University of Medical Sciences. The demographic characteristics of the participants are summarized in Table 1 (Basic characteristics of the respondents).

**Table 1:** Basic characteristics of the respondents (*n* = 41 participants).

	**Mean ±SD**
Age (years)	38.5 ± 13.3
BMI (kg/m^2^)	25.8 ± 5.4
HDRS	19.2 ± 5.9
MMSE	28.9 ± 1.4

### Evaluation of VO_2_max—Astrand-Ryhming Test

As the exercise load increases, the body's oxygen consumption increases proportionally until it reaches a certain value above which the oxygen uptake remains constant despite the increasing load. This value is called the maximal oxygen uptake or maximal aerobic capacity (VO_2_max) and is expressed in liters/minute or milliliters/kilogram of body weight/minute. Aerobic capacity was assessed with a modified Astrand-Ryhming protocol for predicting VO_2_max using a Kettler DX1 Pro cycloergometer (Ense-Parsit, Germany), and HR was monitored using a Polar A-5 pulse meter (Polar Electro Oy, Kempele, Finland). The participants were asked to maintain a constant cadence of 60 revolutions per minute. The trial was stopped when the patient reached the expected heart rate, and it remained stable between 130 and 170 bpm (the difference in heart rate was no more than 5 bpm). The predicted VO_2_max was read from the nomogram or the accompanying tables (43) and multiplied by both the Astrand and the von Dobeln age correction factors. These two predictions in L/min were then converted to mL/kg/min.

### Blood Assays—BDNF, proBDNF, TrkB, and NGFR

A 10-ml sample of venous blood was collected from each patient into an anticoagulant-free tube between 7:30 and 9:30 a.m. after overnight fasting. After 1 h of incubation, the serum was separated by centrifugation, aliquoted, and stored at −70 until the time when all the samples were analyzed. Another blood sample was collected 10 min after the completion of exercise and subjected to the same procedures. Enzyme-linked immunosorbent assay analyses were performed using DuoSet ELISA Development Kits (BDNF cat. no. DY248, proBDNF cat. no. DY3175, TrkB cat. no. DYC397-5, NGFR cat. no. DY367) from R&D Systems (Minneapolis, MN, USA) in accordance with the manufacturer's instructions (with minor modifications). The plates were coated with capture antibodies overnight at room temperature, then washed 3 times and blocked with 1% BSA/PBS for 3 h. The serum samples were diluted in ratios of 1:2 (proBDNF, NGFR), 1:40 (TrkB), and 1:100 (BDNF) in reagent diluent to fit the linear range of standard curves for each analyzed protein. The plates were incubated with 100 μL of samples or standards overnight at room temperature. All the samples and standards were run in duplicates. The detection steps were performed in strict accordance with the manufacturer's instructions. The standard curve ranges were 1,000–15.6 pg/ml (BDNF, TrkB), 3,000–46.8 pg/ml (proBDNF), 2,500–39.06 pg/ml (NGFR). The intra-assay and inter-assay coefficients of variability (CV) were <5 and <10%, respectively.

### Assessment of Cognitive Control—The Stroop Color-Word Interference Test

The Stroop Color-Word Interference Test (SCWT) is often used in psychiatry and neurology to assess primarily cognitive control. The version of the test used in this study consists of two parts: A and B. In part A the participant is asked to read as quickly as possible a list of color names printed in black (achromatic color-word reading). In part B of the test, the participant is presented with the same words, but each word is printed in a color that is inconsistent with the word's meaning—the task is to name the font colors in which the individual words are printed (chromatic color-word reading). The test evaluates the time required to perform part A (treated primarily as a measure of reading speed) and the time required to perform part B—which is a value that takes into account both the speed of reading and the time necessary to activate complex mental processes, including cognitive control and inhibition of automatic verbal responses. Naming font colors that are inconsistent with the meaning of the printed words has been observed to take longer; this phenomenon is known as the Stroop interference effect (44). The number of errors, made primarily in the second part of the test, is also assessed. Each instance of beginning to utter an incorrect word is counted as an error even if the participant makes a self-correction immediately.

### Testing Procedures

The tests were performed in an indoor gym with external stimuli reduced to a minimum (i.e., no music, no videos). A simple, standardized instruction was provided to each participant. The participants were requested not to eat, drink coffee, or smoke during 2 h before the test. They took their medication as usual. All tasks (with the exception of the Astrand-Ryhming test) were performed on the same day in standardized conditions. First, blood samples were acquired (between 7:30 and 9:30 a.m.). Ten minutes after the blood sample was acquired, each patient completed a questionnaire for mood assessment and completed a Stroop test, after which they started the main exercise. The exercise consisted of a 40 min ride on a cycloergometer (name) with the load set at a level of 70%VO_2_ max, corresponding to the individual anaerobic threshold (AT). This value was determined individually for each patient during the exercise test, which determined the VO_2_max value according to the adopted procedure (Astrand-Ryhming test). The initial load was 25–50W (warm-up); after 5 min the load was gradually (25W/min) increased to the target load, which was maintained for 30 min. The last 5 min of exercise was performed with a load of 25W (cooldown). As in the exercise test, the speed during the exercise session was maintained at 60 RPM. After the end of the exercise session, the patients again completed a mood assessment questionnaire and performed the Stroop test, and a second blood sample was collected after 10 min.

### Anthropometric Assessments

Anthropometric measurements included body weight and height. Body weight was measured using a SECA beam balance scale. Height was measured using a SECA stadiometer. The body mass index (BMI) was then calculated (Table 1).

### Medication Use

We recorded the use of antidepressants, mood stabilizers, and antipsychotics. The patients also used somatic medications. Participants taking beta-blocking agents were excluded due to the influence of these agents on heart rate (HR) response (see Supplementary Material).

### Statistical Analyses

The distribution of the data was analyzed using the Kolmogorov-Smirnov and Lilliefors tests. Non-parametric tests were applied. The Wilcoxon signed-rank test was applied to analyze dependent variables (to check the significance of differences of the concentrations of BDNF, proBDNF, TrkB, NGFR, and the SCWT performance before and after exercise). Spearman's rank correlation coefficient was applied to test the correlation between measurable variables (the concentrations of BDNF, proBDNF, TrkB, NGFR, and the SCWT performance). The significance level was set at *p* < 0.05. The analyses were made using the STATISTICA 13.3 software (StatSoft, Kraków, Poland).

## Results

The study analyzed 41 patients (24 women and 17 men) aged 19 to 63 years (mean age: 38 years). Twenty seven patients were diagnosed with a depressive episode in the course of bipolar disorder, while the remaining (14 patients) had their first depressive episode or suffered from recurrent depressive disorder. The mean calculated value of BMI was 25.8 kg/m^2^. The most common comorbidities included hypertension (7 participants) and hypothyroidism (5 participants). Previous pharmacological treatment was maintained during the study. Depression severity was assessed at baseline with the HDRS: the mean score was 19 points, the lowest score was 9 points, and the highest score was 34 points. Exercise load was determined individually for each patient (according to the protocol described in the methods section). The average exercise load was 109W, the minimum load was 50W, and the maximum load was 175W. No statistically significant changes were observed after the exercise sessions with regard to the concentrations of the analyzed neurotrophic factors (BDNF, proBDNF) or their receptors (NGFR, TrkB). The individual values are presented in Table 2.

**Table 2:** Protein concentrations (BDNF, proBDNF, BDNF, NGF, and TrkB) did not change significantly due to exercise.

	**Before exercise**	**After exercise**	**Wilcoxon test T**	***p***
BDNF (ng/ml)	14.84	14.48	378.00	0.67
proBDNF (ng/ml)	2.62	1.87	287.00	0.86
NGFR (ng/ml)	0.5	0.51	375.00	0.83
TrkB (ng/ml)	22.51	21.79	427.00	0.96

Cognitive control was evaluated using the Stroop test. The SCWT assesses inhibitory control of a habitual response in a conflict situation (45) and the ability to switch to a previously unused response criterion (46). The primary indicator described in the Stroop test is the so-called interference effect, i.e., the increase in reaction time when the subject has to react according to a new criterion, different from the one used in the first part of the task. The SCWT evaluates working memory, the efficiency of concentrating attention, and the ability to maintain of executive control (equated with resistance to interference). The test was completed twice by 34 participants—immediately before (SCWT_1_) and about 10 min after exercise (SCWT_2_). Both the mean reading time (29.3 s vs. 47.8 s) and the color naming time (36.7 s vs. 50.7 s) increased. The patients made more mistakes after physical exercise, both in part A (0.2 vs. 1.5) and B (0.6 vs. 1.5). However, the so-called interference effect decreased—the difference between naming and reading times was smaller after exercise (6.2 s vs. 2.4 s). The results are presented in Table 3. No significant correlations were found between the concentrations of the studied neurotrophic factors and the Stroop test results. The correlations are presented in Table 4 (before exercise) and Table 5 (after exercise).

**Table 3:** The table shows the time (in seconds) to complete the individual parts of the Stroop test (SCWT A—version A, monochrome and SCWT B—version B, color), the number of errors (in versions A and B), as well as the interference time (in seconds) before and after exercise.

	**Before exercise**	**After exercise**	**Wilcoxon test T**	***p***
SCWT A (time in s)	29.3 ± 9.3	47.8 ± 21.5	34.00	*p* < 0.0001
SCWT A (number of errors)	0.2 ± 0.6	1.5 ± 2.1	0.00	*p* < 0.001
SCWT B (time in s)	36.7 ± 15.6	50.7 ± 16.1	110.50	*p* = 0.001
SCWT B (number of errors)	0.6 ± 1.1	1.5 ± 1.7	37.50	*p* = 0.004
Interference time (s)	6.2 ± 13.2	2.4 ± 12.6	56.50	*P* < 0.001

*The time of performing individual tasks and the number of errors made have increased significantly, while the time of interference has been significantly shortened*.

**Table 4:** The table shows correlations between the Stroop test results (SCWT A—version A, monochrome and SCWT B—version B, color) and the concentrations of the studied neurotrophic factors before exercise.

		**Rs**	***p***
BDNF	SCWT A (time in s)	−0.226	0.2065
	SCWT A (number of errors)	0.243	0.1728
	SCWT B (time in s)	−0.041	0.8229
	SCWT B (number of errors)	0.224	0.2095
	Interference time (s)	0.142	0.4302
proBDNF	SCWT A (time in s)	0.026	0.8975
	SCWT A (number of errors)	0.058	0.7752
	SCWT B (time in s)	−0.036	0.8570
	SCWT B (number of errors)	−0.087	0.6646
	Interference time (s)	−0.041	0.8404
NGFR	SCWT A (time in s)	−0.068	0.7063
	SCWT A (number of errors)	0.110	0.5410
	SCWT B (time in s)	0.101	0.5748
	SCWT B (number of errors)	0.216	0.2276
	Interference time (s)	0.272	0.1255
TrkB	SCWT A (time in s)	0.198	0.2618
	SCWT A (number of errors)	0.195	0.2695
	SCWT B (time in s)	0.312	0.0720
	SCWT B (number of errors)	−0.034	0.8498
	Interference time (s)	0.170	0.3364

*No significant correlations were found*.

**Table 5:** The table shows correlations between the Stroop test results (SCWT A—version A, monochrome and SCWT B—version B, color) and the concentrations of the studied neurotrophic factors after exercise.

		**Rs**	***p***
BDNF	SCWT A (time in s)	0.235	0.1875
	SCWT A (number of errors)	0.250	0.1607
	SCWT B (time in s)	0.189	0.2930
	SCWT B (number of errors)	0.019	0.9166
	Interference time (s)	−0.070	0.6976
proBDNF	SCWT A (time in s)	−0.031	0.8774
	SCWT A (number of errors)	−0.045	0.8234
	SCWT B (time in s)	−0.214	0.2829
	SCWT B (number of errors)	−0.203	0.3093
	Interference time (s)	−0.126	0.5311
NGFR	SCWT A (time in s)	−0.298	0.0916
	SCWT A (number of errors)	−0.179	0.3185
	SCWT B (time in s)	−0.165	0.3588
	SCWT B (number of errors)	0.131	0.4683
	Interference time (s)	0.339	0.0536
TrkB	SCWT A (time in s)	0.084	0.6370
	SCWT A (number of errors)	0.081	0.6492
	SCWT B (time in s)	0.083	0.6392
	SCWT B (number of errors)	−0.203	0.2494
	Interference time (s)	−0.104	0.5600

*No significant correlations were found*.

## Discussion

Physical activity is used also as one of the strategies to maintain (or improve) cognitive function. Researchers continue to investigate the mechanism of this phenomenon. The impact of physical activity on the cognitive abilities of depressed patients is unclear. It has also not been clearly established whether a single session of physical activity can change the concentration of neurotrophic factors.

As a result of the study conducted by our team, we did not observe statistically significant changes after exercise with regard to the concentrations of the analyzed proteins (BDNF, proBDNF, NGFR, TrkB). Similar results were obtained in some of the studies presented in the introduction. However, they mainly concerned the concentration of BDNF. According to the authors' knowledge, the available literature does not contain much data on the changes in proBDNF, TrkB and NGRF concentrations under the influence of physical activity in patients with an episode of depression. In our study, these values did not change significantly. However, many publications indicate that, under the influence of exercise, the concentration of BDNF increases (28, 29). This observation usually applies to healthy individuals (30, 31, 47).

Most of the research to date has focused on exercise programs consisting of multiple sessions. Regular exercise is likely to have a greater impact on neuroplasticity, which may also result in sustained improvement in cognitive function. Fewer studies are available on the impact of a single bout of physical activity on cognitive performance and change in the concentrations of selected neurotrophic factors in depressed patients. Contrary to our experience, some of them showed both an increase in the concentration of neurotrophic factors and an improvement in cognitive functions (32, 42). Studies linking physical activity and cognitive function have been conducted in healthy populations. The results indicate a positive effect of exercise on cognitive function. The less beneficial effect we observed among the depressed population may be related to several factors. Most often, these individuals have a sedentary lifestyle and are reluctant to engage in complex activities. The baseline BDNF level in patients suffering from depression was found to be lower compared to healthy subjects. In reviewed studies, men were often a significant proportion of participants, and a greater increase in BDNF was observed in the male population than in the female population. In our project, women, who are diagnosed with depression several times more frequently, were predominant.

The obtained results may also be related to the selected exercise intensity. The studies suggest that exercise load may differentiate the influence of physical activity on cognitive functions (38, 41). In many studies, however, a moderate intensity (this was used in our experiment) was enough to obtain the desired effects.

It is suggested that SCWT can assess various cognitive functions (48). The Stroop test measures primarily speed of visual search (part A—reading), working memory, and conflict monitoring (part B—naming colors) (49, 50). It is also used to measure other cognitive functions such as attention, processing speed, cognitive flexibility (51). As shown in previous publications, these functions in patients with depression are reduced compared to healthy subjects (2–7). In the study by Basso and Suzuki (40), they indicate that physical activity can affect attention, working memory, decision making, and inhibitors control, which contributed to the choice of SCWT in our study. The Stroop test was repeated twice in quick succession. Improvement in interference time that we observed may be due to practice effects. It is wondering, why the other variables (time and number of errors) would not also improve. It is possible that this is caused by fatigue after exercise.

### Implications and Limitations of the Study

Our study has several limitations. It was conducted on a small group of participants. The study did not include a control group. It enrolled patients with both BD and MDD. It is believed that the baseline level of cognitive function and the concentrations of selected neurotrophic factors in both groups may differ significantly. In order to exclude significant cognitive deficits, the patients were examined by MMSE during recruitment, and they all scored above 27 points. It was not possible to confidently estimate the level of the subjects' physical activity before hospitalization; this estimation relied on the self-assessment of the participants (most declared a complete lack or low levels of exercise at least since the onset of depression). Differences in motivation and commitment to task performance were evident and attributable, at least partly, to the presence and severity of depressive symptoms. Notwithstanding, all participants were subject to the same procedures and verbal instructions and cues for each stage of the test. The influence of medication on both physical performance and neurocognitive abilities was not without significance. Blood sample acquisitions were performed at two time points. It is conceivable that a change in the concentration of the examined proteins would be observed during exercise or after a longer period of time after its completion. For the assessment of selected cognitive functions, we used only the Stroop test, which measures specific abilities. This test was repeated twice in a short time, which could activate the so-called practice effect. In order to objectify the results, it is necessary to extend the battery of tests and include a control group. We believe that it would be worthwhile to continue the research in this area and to eliminate the limitations listed above.

## Conclusion

No change in the concentrations of selected neurotrophic factors and receptors after a single bout of exercise was observed in this study. Neither were there any significant correlations between the concentrations of BDNF, proBDNF, TrkB, NGFR and the SCWT performance. The opposing results from previous studies indicate the complexity of the problem and suggest that the mechanism linking physical activity, depression, and cognitive function is a multifactorial one. Understanding it could contribute to the creation of a therapeutic exercise program whose effectiveness would be supported by scientific evidence.

## Data Availability Statement

The original contributions presented in the study are included in the article/Supplementary Material, further inquiries can be directed to the corresponding author/s.

## Ethics Statement

The studies involving human participants were reviewed and approved by Ethical Committee of the Poznan University of Medical Sciences. The patients/participants provided their written informed consent to participate in this study.

## Author Contributions

FR and AJ-M were involved in the conceptualization and design of the study, analysis, and interpretation of data. AJ-M, KDr, and KDo acquired data. AJ-M, KDr, KDo, MK, KJ, and GK were involved in the conduct of the study. MS performed protein determinations and statistical analysis. AJ-M wrote the first draft of the article. FR supervised the study and revised the manuscript. All authors contributed to the article and approved the submitted version.

## Conflict of Interest

The authors declare that the research was conducted in the absence of any commercial or financial relationships that could be construed as a potential conflict of interest.

## Publisher's Note

All claims expressed in this article are solely those of the authors and do not necessarily represent those of their affiliated organizations, or those of the publisher, the editors and the reviewers. Any product that may be evaluated in this article, or claim that may be made by its manufacturer, is not guaranteed or endorsed by the publisher.
