# Supplementary material for: The Effect of Physical Activity on Neurotrophin Concentrations and Cognitive Control in Patients With a Depressive Episode
Source: Front Psychiatry. 2022 Apr 25;13:777394. doi: 10.3389/fpsyt.2022.777394 (PMC9084496; doi:10.3389/fpsyt.2022.777394)
Supplement: Supplementary file 1 [file Table_1.pdf]

Supplementary material. Drugs used by study participants.

Types of medications	Number of participants
**Antidepressants:**	
venlafaxine	13
mirtazapine	5
paroxetine	5
sertraline	5
fluoxetine	4
duloxetine	3
reboxetine	3
bupropion	1
escitalopram	1
trazodone	1
**Mood stabilizers:**	
lithium	17
lamotrygine	10
valproic acid	5
carbamazepine	4
**Neuroleptics:**	
quetiapine	21
olanzapine	5
risperidone	4
clozapine	2
aripiprazole	1
**Others**	
pregabalin	2
topiramate	1
methylphenidate	1
levothyroxine	4
amlodipine	3
ramipril	2
doxazosin	1
rosuvastatin	2
metformin	1

omeprazole	1
azathioprine	1
